# Plasma metabolomics study reveals the critical metabolic signatures for benzene-induced hematotoxicity

**DOI:** 10.1172/jci.insight.154999

**Published:** 2022-01-25

**Authors:** Xiaoli Guo, Lei Zhang, Jingyu Wang, Wei Zhang, Jing Ren, Yujiao Chen, Yanlin Zhang, Ai Gao

**Affiliations:** 1 Department of Occupational Health and Environmental Health, School of Public Health, and; 2 Beijing Key Laboratory of Environmental Toxicology, Capital Medical University, Beijing, China.; 3 Research Center of Occupational Medicine, Peking University Third Hospital, Haidian District, Beijing, China.

**Keywords:** Metabolism, Bone disease, Fatty acid oxidation, Leukemias

## Abstract

Metabolomics has been used to explore the molecular mechanism and screen biomarkers. However, the critical metabolic signatures associated with benzene-induced hematotoxicity remain elusive. Here, we performed a plasma metabolomics study in 86 benzene-exposed workers and 76 healthy controls, followed by a validation analysis in mice, to investigate the dynamical change of the metabolic profile. We found that 8 fatty acids were significantly altered in both benzene-exposed worker and benzene-exposed animal models. These metabolites were significantly associated with S-phenylmercapturic acid and WBC, and they mediated the benzene-induced WBC decline. Furthermore, in vivo results confirm that fatty acid levels were dynamically altered, characterized by a decrease at 15 days and then sharp increases at 30 and 45 days. Following these identified fatty acids, the potential metabolic pathways were investigated. Fatty acids, as precursors for fatty acid oxidation, may disturb the balance of fatty acid biosynthesis and degradation. Our results reveal that fatty acid metabolism was strongly reprogrammed after benzene exposure. This abnormal change of fatty acids might be the key metabolic signature associated with benzene-induced hematotoxicity.

## Graphical abstract

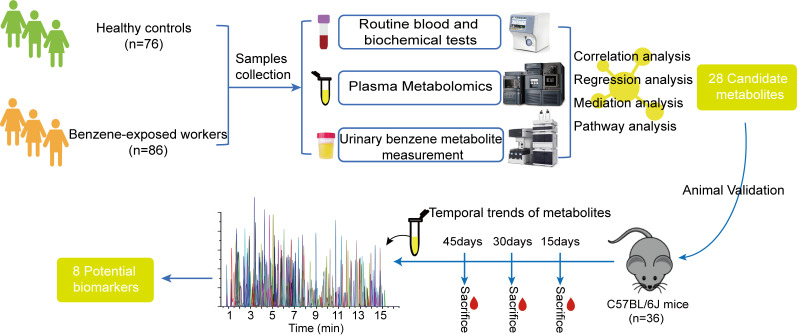

## Introduction

Benzene continues to be a nonnegligible contributor to leukemia in occupationally and environmentally exposed populations. Recent epidemiological studies have shown that global acute myelocytic leukemia (AML) morbidity and mortality have been increasing over the past 28 years and that occupational exposure to benzene is considered a major risk factor (1). According to the latest report from the National Bureau of Statistics, China’s pure benzene output has been increasing year after year, which may partly explain why the benzene poisoning and leukemia cases have remained high in recent years (2). Previous studies have shown that workers exposed to 1 ppm benzene exhibited no substantial WBC harm, but their BM hematopoietic capability was severely reduced in terms of renewal and differentiation (3). This result suggested the limitations of a single WBC indicator for diagnosing hematologic adverse events caused by low-level benzene exposure. Thus, discovering potential biomarkers to identify high-risk individuals earlier is crucial for developing effective strategies to prevent chronic benzene poisoning and leukemia.

Metabolomics-based strategies to characterize the toxicity of environmental pollutants and the risk of disease have become a hot topic of research (4, 5). However, information on the impact of benzene exposure on the human metabolome is extremely limited. Results from a cross-sectional study based on untargeted plasma metabolomics reveal that 41 metabolic pathways were significantly altered between benzene-exposed workers (*n* = 33) and healthy controls (*n* = 25); these pathways included carnitine shuttle, fatty acid metabolism, and glycolysis. This may be attributable to mitochondrial dysfunction (6). Wang et al. also evaluated the effects of exposure to environmental heavy metals, polycyclic aromatic hydrocarbons (PAHs), and benzene on urinary metabolites in Chinese children and the elderly. Although benzene exposure increased more than heavy metals and PAHs in the exposure group, no correlation was found between S-phenylmercapturic acid (SPMA) and urinary metabolites (7). Inhaled benzene is converted into benzoquinone or hydroquinone, and these metabolites destroy human blood components. However, only 0.005%–0.3% of benzene is metabolized to produce SPMA (8), and it is excreted from the urine. This metabolic feature may partly explain the insignificant correlation between benzene and urine metabolites in the above study.

Metabolomics focuses on the identification of promising biomarkers associated with various diseases or environmental exposures (9). Recently, it was shown that a human plasma metabolic phenotype allowed to successfully discriminate cancer types (10). Previous studies have revealed that certain metabolites and metabolic enzymes were associated with the risk of leukemia (11, 12). A case-control study including 400 AML patients and 446 healthy controls has shown that the glucose metabolism was modified in AML patients; furthermore, 6 metabolites (pyruvic acid, glycerol-3-phosphate, 2-oxoglutarate, 2-HG, lactate, and citrate) demonstrated prognostic value in cytogenetically normal AML patients (11). To identify whether leukemia patients treated with tyrosine kinase inhibitors have adverse cardiovascular effects, Caocci et al. found that 15 metabolites were significantly enriched in leukemia patients who developed cardiovascular injury (13). These studies provided further insights into the underlying etiology and pathology of leukemia.

Before the development of hematological malignancies, benzene-exposed populations experienced long-term hematologic toxicity characterized by decreased blood cells (14). To explore the molecular mechanism and screen biomarkers of benzene-induced hematotoxicity, the variation of endogenous metabolites in urinary, BM cells, and plasma of male C3H/He mice exposed to benzene was detected using untargeted metabolomics (15, 16). These studies have revealed a significant difference in the metabolic profiles between benzene-exposed mice and controls (15). Up to now, few articles have touched upon metabolic changes in low-level benzene–exposed workers. We speculate that endogenous metabolites are potential biomarkers for chronic benzene poisoning and leukemia, and they play important roles in mediating benzene-induced hematotoxicity. Furthermore, metabolite levels could shift with worsening of benzene-induced hematotoxicity.

**Figure 1:**
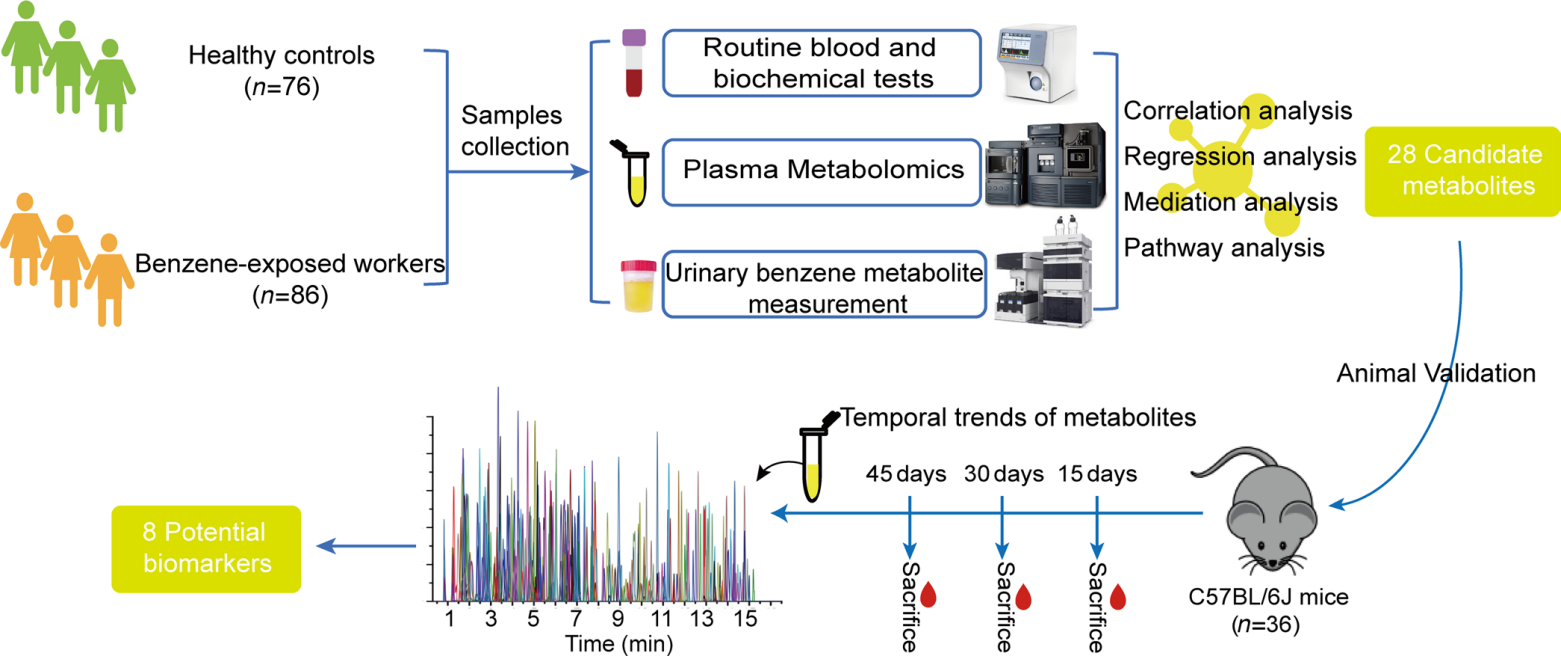
Plasma metabolomics study design in humans (*n* = 162) and mice (*n* = 36) for revealing biomarkers associated with early hematotoxicity from benzene exposure.

Here, absolute quantitative metabolomics (17) was applied to analyze plasma metabolite changes in 86 low-level benzene–exposed workers and 76 healthy controls, followed by a validation analysis in mice exposed to benzene for 15, 30, and 45 days to identify and confirm the dynamically changing metabolic profile (Figure 1). Based on the identified differential metabolites, the relevant networks and metabolic pathways were investigated. This work reveals the critical metabolic signatures in benzene-induced hematotoxicity and provides a panel of potential effector molecules for early health screening for benzene exposure.

## Results

### General information for participants.

A total of 162 subjects aged from 20–50 years were recruited in this study, including 86 low-level benzene–exposed workers and 76 healthy controls. The participants’ characteristics and baseline data are presented in Table 1. There were no significant differences in terms of sex, BMI, and smoking and alcohol status between 2 groups. The average age of benzene-exposed workers was lower compared with controls (*P* < 0.05).

### Benzene exposure and health effect indicators.

To conduct the benzene exposure assessment, the levels of SPMA, trans,trans-muconic acid (tt-MA), 2-methylhippuric acid (2-MHA), and 3-MHA — classic internal exposure biomarkers — were measured in the urine of benzene-exposed workers and healthy controls based on an ultraperformance liquid chromatography coupled with tandem mass spectrometry (UPLC-MS/MS) detectors (Supplemental Table 1; supplemental material available online with this article; https://doi.org/10.1172/jci.insight.154999DS1). We found that differences in urinary SPMA and tt-MA levels were significantly identified in both groups, with SPMA being significantly elevated in benzene-exposed workers (*P* < 0.05), suggesting that SPMA might be a sensitive marker of low-level benzene exposure. In addition, the results of the sex-stratified analysis showed a 4.6-fold increase in median levels of SPMA in male benzene-exposed workers relative to controls, but no significant difference was shown in female workers (*P* < 0.01). The levels of the health effect indicators of benzene exposure, including whole blood cells and liver function indicators, were subsequently analyzed. As seen from Supplemental Table 1, the blood cell counts were predominantly within normal ranges, but the level of WBC, a conventional blood biomarker, in benzene-exposed workers was lower compared with healthy controls (*P* < 0.05), and this declining trend was more pronounced in male workers (*P* < 0.01). In female workers, we only found that the hemoglobin (HGB) level was higher than that in control group (*P* < 0.05), and alanine transaminase (ALT), a sensitive marker of hepatocellular damage, was also remarkably increased in benzene-exposed female workers with the mean value of 22.50 U/L.

### Plasma metabolic profiles in low-level benzene–exposed worker.

**Figure 2:**
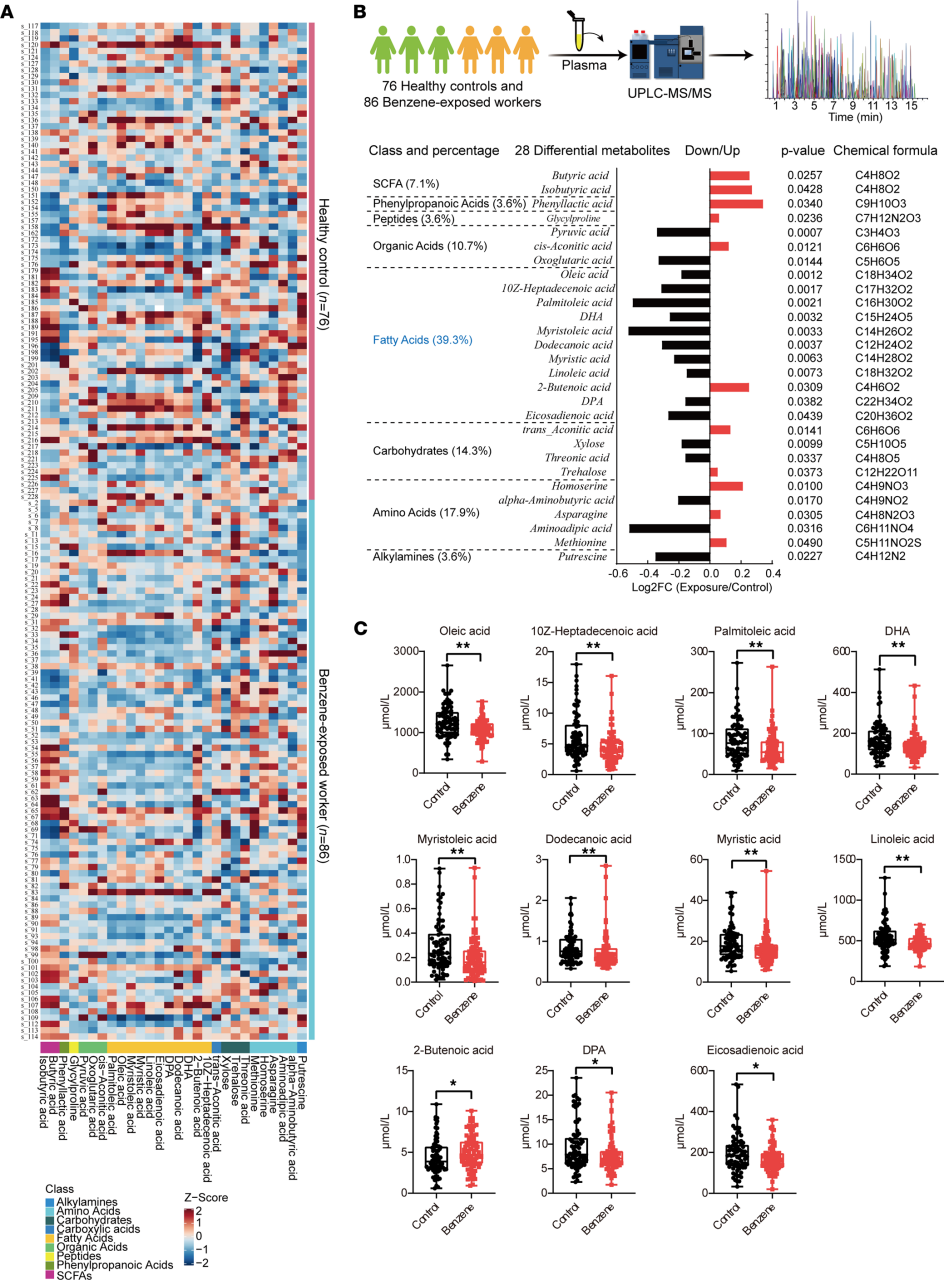
Plasma metabolic profiles in benzene-exposed workers (*n* = 86) and healthy controls (*n* = 76). (**A**) Visualization of 28 plasma differential metabolite expression values in 2 groups by heatmap. (**B**) Classification and expression changes of differential metabolites. (**C**) Median concentrations of the 11 screened plasma fatty acid levels. Each plasma sample was measured 3 times based on UPLC-MS/MS. Mann-Whitney *U*/Wilcoxon rank-sum test was performed to compare the differences in metabolite levels between the 2 groups. *P < 0.05; **P < 0.01.

To investigate potential metabolites involved in benzene-induced hematotoxicity, the quantification metabolomics was conducted in 86 low-level benzene–exposed workers and 76 healthy controls. A total of 300 metabolites were identified by UPLC-MS/MS. Twenty-eight differential metabolites (including 17 downregulated and 11 upregulated) were obtained as potential biomarkers between the benzene-exposed workers and controls under the *P* < 0.05 (Figure 2, A and B, and Supplemental Table 2). These metabolites were mainly originated from the fatty acids (39.3%) (oleic acid, 10z-heptadecenoic acid, palmitoleic acid, DHA, myristoleic acid, dodecanoic acid, myristic acid, linoleic acid, 2-butenoic acid, DPA, and eicosadienoic acid), while a small number were amino acids (17.9%) (homoserine, α-aminobutyric acid, asparagine, aminoadipic acid, methionine, and putrescine), carbohydrates (14.3%) (trans-aconitic acid, xylose, threonic acid, and trehalose), organic acids (10.7%) (pyruvic acid, cis-aconitic acid, and oxoglutaric acid), short-chain fatty acids (SCFAs) (7.1%) (butyric acid, isobutyric acid), phenylpropanoic acids (3.6%) (phenyllactic acid), peptides (3.6%) (glycylproline), and alkylamines (3.6%) (putrescine). Furthermore, 11 fatty acids expressed a consistent downward trend (Figure 2C), suggesting that abnormal fatty acid changes were likely to play important roles in benzene-induced hematotoxicity.

### Altered plasma metabolites in mice exposed to benzene for 15, 30, and 45 days.

**Figure 3:**
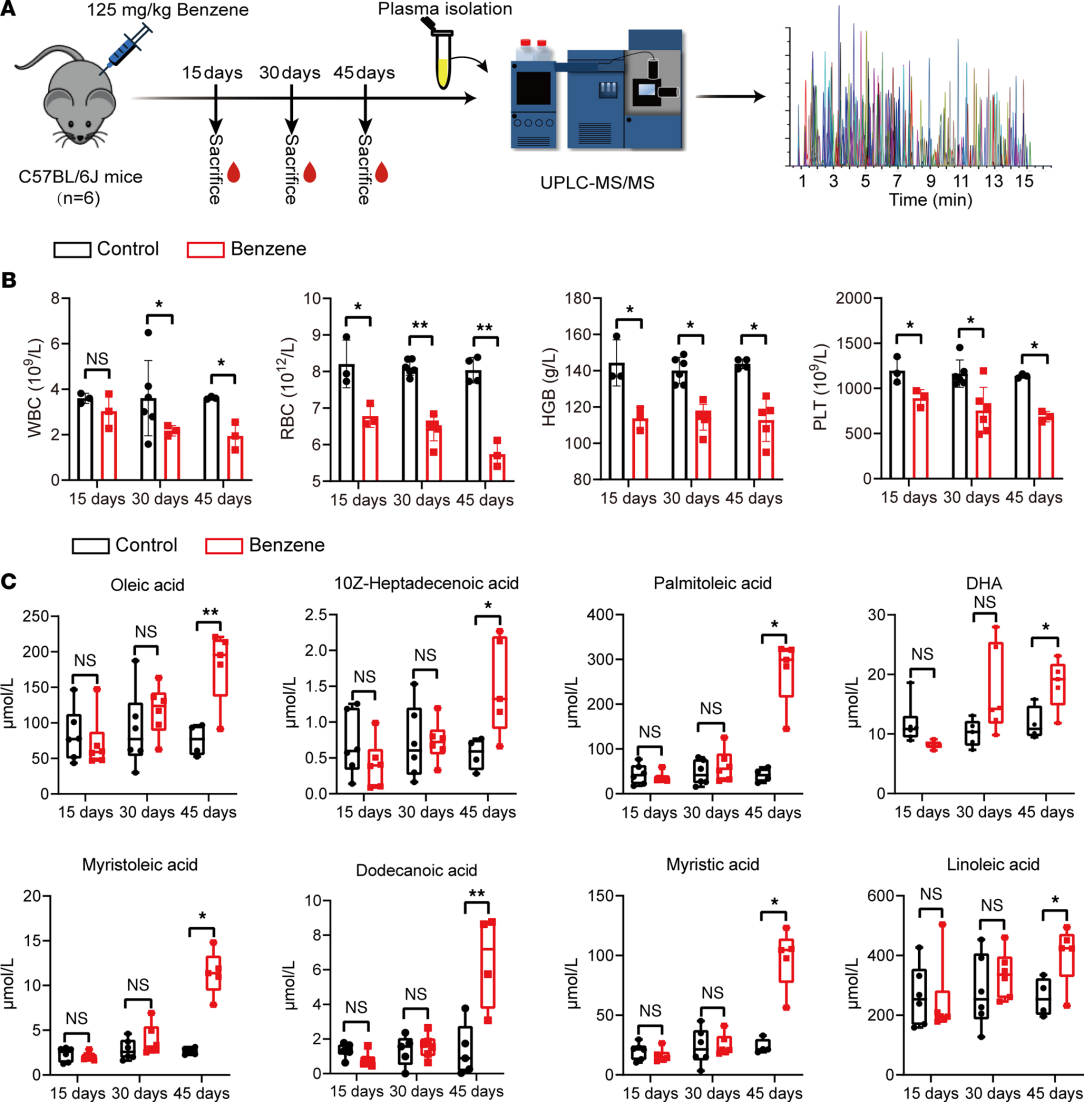
Plasma metabolic profiles in mice exposed to benzene for 15, 30, and 45 days. (**A**) Animal experiment design diagram. (**B**) Changes in hematological parameters in mice exposed to benzene for 15, 30, and 45 days (*n* = 3-6 for per group). (**C**) The concentration and temporal trends of identified coplasma metabolites in mice after 15, 30, and 45 days of benzene exposure (*n* = 6 for per group). Each sample was measured 3 times for routine blood testing and UPLC-MS/MS analysis. Student’s *t* test was performed to compare blood cell levels between the 2 groups, and metabolite levels were compared using the Mann-Whitney *U*/Wilcoxon rank-sum test. *P < 0.05; **P < 0.01.

The factors influencing endogenous metabolite levels in a population are complicated. In addition, changes in metabolite levels and disease progression are closely related. With these factors in mind, we performed the same metabolomic analysis with absolute quantification in background-consistent C57BL/6J mice to further validate the association between metabolic alterations and benzene-induced hematotoxicity. In our study, mice were treated with 125 mg/kg of benzene for 15, 30, and 45 days by s.c. injection (Figure 3A). To begin with, there were no statistical differences in the WBC levels in mice at 15 days of benzene exposure. With increasing exposure time, the whole blood cells in the benzene-exposed group were significantly decreased compared with controls at 30 and 45 days (Figure 3B). Histological analysis also displayed no significant disturbance to BM cells of the mice at 15 days of benzene exposure, which manifested neatly arranged, regulated, and satiated ellipses. Administration of benzene at 125 mg/kg at 30 and 45 days, noticeable cell loss, cell swelling, and aggregated fat droplets were visible (Supplemental Figure 1). These data indicate that benzene could induce hematotoxicity in a time-dependent manner and that administration of benzene at 125 mg/kg with 45 days of exposure could cause remarkable hematotoxicity.

The full quantitative targeted metabolomics was used to analyze the plasma levels in mice after 15, 30, and 45 days of benzene exposure. Our results reveal that there were 8 fatty acids (oleic acid, 10z-heptadecenoic acid, palmitoleic acid, DHA, myristoleic acid, dodecanoic acid, myristic acid, linoleic acid) in mouse plasma, consistent with the benzene-exposed workers. However, we found that the levels of the fatty acid showed a slight decrease at the 15 days and an increase from the 30 days, with a significant difference at 45 days (Figure 3C). This finding suggested that early exposure to benzene could cause a slight decrease in fatty acid levels. However, with the extension of exposure time, the hematopoietic toxicity was aggravated, and the metabolism of mice became progressively disturbed and decompensated.

### Associations of plasma fatty acids with SPMA and WBC.

**Figure 4:**
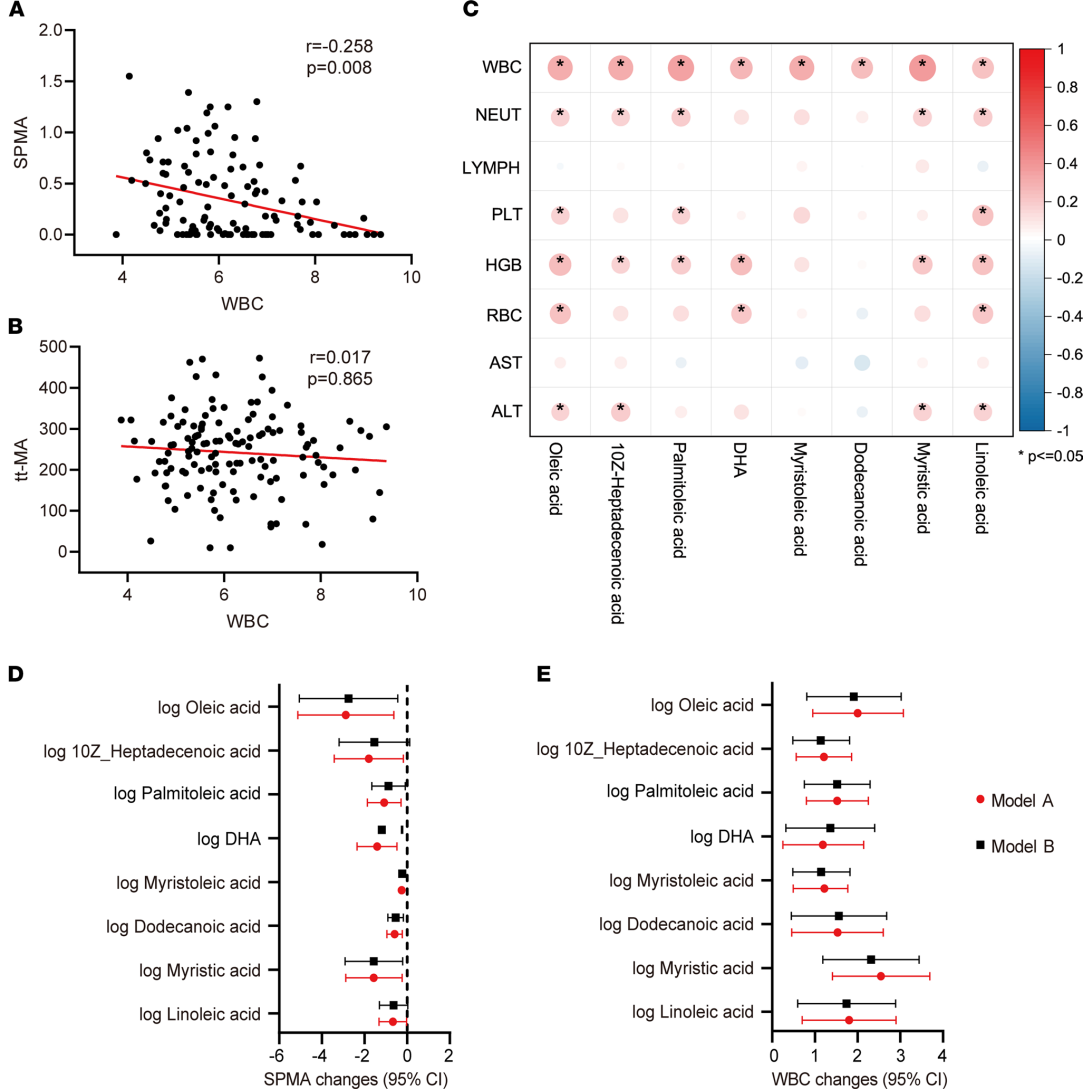
Associations among plasma fatty acids, WBC, and urinary SPMA in all participants (*n* = 162). (**A** and **B**) Correlation analysis of WBC and urinary benzene metabolites (SPMA and tt-MA) after adjusting for age, sex, BMI, and smoking and alcohol status. (**C**) Correlation of WBC with plasma fatty acids. *P < 0.05. (**D** and **E**) Regression analysis of plasma fatty acids with WBC and SPMA. Model A: crude (unadjusted for age, sex, BMI, smoking, and alcohol status); Model B: adjusted for age, sex, BMI, smoking and alcohol status. To adjust to the same order of magnitude, the estimated values (β) and 95%CI of oleic acid and linoleic acid were divided by 100, and DHA and palmitoleic acid were divided by 10.

Currently, alterations in WBC are an important diagnostic basis for benzene-caused hematologic disorders. Thus, the associations among differential fatty acids, urinary benzene metabolites, and WBC were analyzed in this study (Figure 4). After adjusting for age, sex, BMI, and smoking and alcohol status, a decreased WBC was significantly associated with increased urinary SPMA in benzene-exposed workers but not with tt-MA (Figure 4, A and B). A similar strong correlation was also found in male subjects according to the results of the stratified analysis (Supplemental Table 3). In addition, there was a significant positive correlation between WBC and 8 differential fatty acids in subjects (Figure 4C), and a similarly strong correlation was also presented in animal experiments (Supplemental Figure 2).

To better understand the relationship between abnormal plasma fatty acids and benzene-induced hematotoxicity, we further analyzed the associations of differential fatty acids with SPMA and WBC by a linear regression model. After adjusting for confounding factors (age, sex, BMI, and smoking and alcohol status), plasma fatty acids were negatively correlated with SPMA, except for 10z-heptadecenoic acid and linoleic acid (Figure 4D). Besides, 8 fatty acids presented a significant positive correlation with WBC in all participants (Figure 4E and Supplemental Table 4), and this is consistent with the results of the Spearman analysis shown in Figure 4C. These data reinforce that these fatty acids might act as key mediators of benzene-induced hematotoxicity.

### Mediation analysis on differential plasma metabolites in response to benzene exposure.

Before the hematologic malignant transformation, benzene-exposed workers experienced long-term hematologic toxicity characterized by a decline in WBC in the early stages of benzene exposure and a decline in whole blood cells in severe situations. To examine whether the relationship between benzene exposure and the decreased WBC was mediated by differential plasma metabolites, simple mediation models were applied in this study (Table 2). Expectedly, 8 fatty acids (oleic acid, 10z-heptadecenoic acid, palmitoleic acid, DHA, myristoleic acid, dodecanoic acid, myristic acid, and linoleic acid) as complementary mediators participated in the decrease of WBC in association with benzene exposure.

### The interrupted fatty acid metabolic network involved in benzene-induced hematotoxicity.

**Figure 5:**
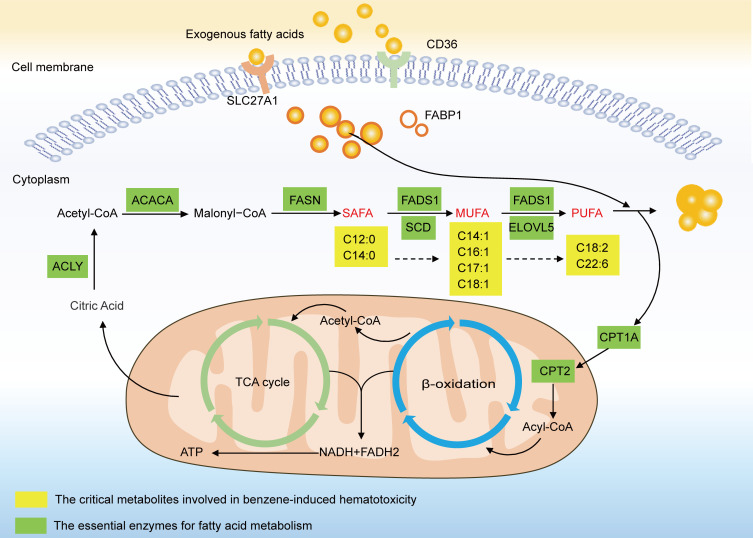
Interrupted fatty acid metabolism associated with benzene-induced hematotoxicity. SAFA, saturated fatty acid (no unsaturated double bonds); MUFA, monounsaturated fatty acid (1 unsaturated double bond); PUFA, polyunsaturated fatty acid (≥2 unsaturated double bonds); C12:0, dodecanoic acid; C14:0, myristic acid; C14:1, myristoleic acid; C16:1, palmitoleic acid; C17:1, 10z-heptadecenoic acid; C18:1, oleic acid; C18:2, linoleic acid; C22:6, DHA; CD36, CD36 molecule; SLC27A1, solute carrier family 27 member 1; FABP1, fatty acid binding protein 1; ACACA, acetyl-CoA carboxylase α; FASN, fatty acid synthase; FADS1, fatty acid desaturase 1; SCD, stearoyl-CoA desaturase; ELOVL5, ELOVL fatty acid elongase 5; CPT1A, carnitine palmitoyltransferase 1A; CPT2, carnitine palmitoyltransferase 2; NADH, nicotinamide adenine dinucleotide; FADH2, flavine adenine dinucleotide, reduced; and ACLY, ATP citrate lyase.

Dysregulation of fatty acid metabolism has been associated with a variety of diseases and is emerging as an important player in leukemia. Both KEGG- and MSEA-based network analyses (https://www.metaboanalyst.ca/) displayed a significant enrichment of pathways related to fatty acid metabolisms, such as fatty acid biosynthesis, β-oxidation of fatty acid, and carnitine transport (Supplemental Figure 3). These data show that benzene might disrupt fatty acid metabolism to induce hematotoxicity. Herein, we established an aberrant metabolic correlation network related to benzene exposure based on the above results and the KEGG database of metabolic pathways (https://www.kegg.jp/kegg/pathway.html) (Figure 5). Fatty acid molecules pool with different degrees of saturation are formed and undergo complicated steps of synthesis, desaturation, and elongation by the action of metabolism-critical enzymes. Some of these processed fatty acids are stored in the fat, liver, and muscle tissue in the form of lipid droplets, and others are used as raw materials for combustion to participate in fatty acid oxidation (FAO). Combining the results from the population and animals in this study, there was a slight decrease in plasma fatty acid levels in the early exposure to benzene, but the increase in blood toxicity was accompanied by a sustained increase in fatty acids as the days of benzene exposure increased. These results support that benzene certainly disturbed fatty acid metabolism, and this alteration was associated with the degree of hematotoxicity. The mechanisms of fatty acid metabolism involved in benzene-induced hematotoxicity are of great complexity; therefore, more validation work is needed in the future.

## Discussion

Benzene exposure has been well known to result in a decrease in peripheral blood cells and, eventually, benzene poisoning and leukemia (18). Currently, the diagnosis for benzene poisoning is still mainly based on the changes of hemogram, especially WBC counts (19, 20). According to the latest Chinese occupational health standards, a history of benzene exposure with a WBC count < 4 × 10^9^/L is an essential diagnostic criterion for mild benzene poisoning (21). In our study, although the WBC counts in benzene-exposed workers were decreased compared with controls, its ranges (4.07 × 10^9^/L to 8.78 × 10^9^/L) remained within the normal limits for a healthy population (3.50 × 10^9^/L to 9.50 × 10^9^/L). To assess the internal exposure levels of benzene-exposed workers, urinary SPMA — a specific and sensitive biomarker of low-level benzene exposure — has been widely used for biomonitoring and health risk assessment of benzene (22). Some countries, such as the United States (25 μg/g creatinine [Cr]), Germany (40 μg/g Cr), and Singapore (45 μg/g Cr), have accordingly established biological exposure limits for urinary SPMA at 0.5–1 ppm of airborne benzene concentration (23, 24). In this study, we found that the median concentration of urinary SPMA in benzene-exposed workers (0.78 μg/g Cr) was 6.5 times higher than that of the control group (0.12 μg/g Cr), but it was considerably lower than the reported standard limits of urinary SPMA. Additionally, increased levels of urinary SPMA were significantly associated with decreased WBC. These results indicated that the benzene-exposed workers in this study were at low levels of benzene exposure and suffered no significant hematotoxicity. Whereas urinary SPMA was a sensitive biomarker reflecting low levels of benzene exposure, similar results were also found in both animals or populations from previous low-level benzene toxicity studies (25, 26). An epidemiological study confirmed that, at airborne benzene concentrations < 1 ppm, the WBC counts of benzene-exposed workers were within the normal range (4 × 10^9^/L to 10 × 10^9^/L) for clinical diagnosis, but their renewal and differentiation of hematopoietic stem cells (HSCs) had been significantly restrained (3). Therefore, WBC counts, a conventional blood biomarker, provide extremely limited insight in early benzene-exposed population health surveillance and diagnosis.

Long-term chronic benzene exposure is an important driver of the development of hematological malignancies. Although some studies have not found the occurrence of AML or myelodysplastic syndromes (MDS) in benzene-exposed populations, workers exposed to < 5 ppm or even < 1 ppm benzene had statistically significant increases in hematologic toxicity (27, 28). This suggested that the exploration of sensitive biomarkers is of great practical importance for risk assessment and health screening of early blood toxicity in occupational benzene–exposed workers. To characterize the metabolic profile associated with benzene exposure, a cross-sectional study including 86 low-level benzene–exposed workers and 76 healthy controls was conducted for absolute quantitative metabolomics. In our study, 28 plasma metabolites were identified to have differential expression in benzene-exposed workers and healthy controls. These metabolites were mainly originated from the fatty acids, while a small number were amino acids, carbohydrates, organic acids, SCFAs, peptides, and alkylamines. Of the 28 differential metabolites, fatty acids (including oleic acid, 10z-heptadecenoic acid, palmitoleic acid, DHA, myristoleic acid, dodecanoic acid, myristic acid, linoleic acid, 2-butenoic acid, DPA, and eicosadienoic acid) were the most altered metabolic pool, with a 39.3% proportion, and they were significantly downregulated in benzene-exposed workers. Abnormal metabolite levels promote the development of disease. Human metabolite levels are influenced by a combination of multiple lifestyle and environmental exposures; therefore, it is difficult to determine the relationship between metabolite levels and disease. Considering these factors, metabolomic analyses were also performed in mice after 15, 30, and 45 days of benzene exposure to further validate the association between metabolic alterations and benzene-induced hematotoxicity. In an in vivo study, we identified 8 significantly altered fatty acids (oleic acid, 10z-heptadecenoic acid, palmitoleic acid, DHA, myristoleic acid, dodecanoic acid, myristic acid, and linoleic acid), consistent with the metabolic profile of benzene-exposed workers. At 15 days of benzene exposure, we found that there was no significant decrease in WBC in mice, suggesting that mice might be in an early stage of benzene exposure without significant hematotoxicity. This altered blood count was similar to that of the workers exposed to low-level benzene in this study. Similarly, we also found a decrease in fatty acids in mice exposed to benzene for 15 days in the plasma metabolite profiles. Starting from 30 days, a remarkable decrease in whole blood counts occurred in benzene-exposed mice. This was consistent with the results of previous studies (29). Interestingly, we discovered a reverse increase in the levels of fatty acids, with a significant peak at 45 days of benzene exposure. The results of in vivo studies suggest that hematotoxicity in mice was progressively worse with increasing days of benzene exposure. The abnormal fatty acid alterations might be the underlying pathological mechanism of benzene-induced hematotoxicity. Therefore, we hypothesized that early benzene exposure induced a slight decrease in fatty acids levels, while the increase in days of benzene exposure was accompanied by elevated hematotoxicity, which led to a decompensated rise in fatty acids. Furthermore, we found that fatty acids were associated with the decrease of WBC and the increased urinary SPMA. After adjusting age, sex, BMI, and smoking and alcohol status, the results of the mediation analysis showed that the above-mentioned 8 fatty acids mediated the associations between SPMA and WBC. These findings suggest that fatty acids might play an important role in benzene-induced hematotoxicity.

Fatty acids are important components of lipids and are involved in the fluidity of cell membranes and energy storage and supply. Abnormal fatty acid levels were associated with the development of various diseases and the metabolic reprogramming of cancer (30). A metaanalysis based on 17 prospective cohort studies showed that high levels of blood n-3 fatty acids, such as EPA, DPA, and DHA, were associated with a reduced risk of all-cause mortality (31). N-3 polyunsaturated fatty acids (PUFA; such as EPA, DHA) have antiinflammatory and antioxidant potential in a variety of diseases, such as nonalcoholic fatty liver, diabetes, and Alzheimer’s disease (32, 33). However, fatty acids containing different double bonds perform different physiological functions. Herbert et al. reported that dietary saturated fatty acids (SAFA) were a leading risk factor for promoting inflammation in psoriasis, which increases the inflammatory response primarily by increasing myeloid cell sensitivity in response to proinflammatory stimuli (34). In recent years, fatty acid metabolism has attracted more attention in blood-related cancers such as AML. Dysregulation of critical genes (such as ACACA, FASN, and CPT1A) in fatty acid metabolism, leading to an imbalance in fatty acid degradation and production, was considered as a potential metabolic marker for cancer cells (35). However, it is unclear whether the early hematotoxicity caused by benzene has a similar metabolic profile to AML.

Several previous studies have explored the effect of benzene exposure on metabolic changes (15, 16, 36, 37), but they mainly focus on the relative expression detection of plasma metabolites in animals or benzene-exposed workers. Until now, accurate quantitative metabolomics to detect the real level of plasma metabolites and explore associations with urinary benzene metabolites has not been obtained, to our knowledge. In this study, we found that abnormal fatty acid metabolism was the dominant metabolic change of benzene-exposed workers. Fatty acids were shown as complementary mediators to participate in the decreasing of WBC associated with benzene exposure. AML has a high demand for energy production to meet the malignant growth and proliferation of cells. Lipids, an essential energy source, produce NADH and ATP through FAO to maintain the body’s energy balance (30). Dodecanoic acid was known as an intermediate of fatty acids synthesis pathway. The decrease in dodecanoic acid in plasma early in benzene exposure implies an accelerated synthesis of fatty acids, suggesting an increased utilization of fatty acids by the body in association with benzene exposure. Other fatty acids — such as myristoleic acid, palmitoleic acid, DHA, DPA, and oleic acid — were the precursors of FAO (38). The reduction of these fatty acids in early exposure to benzene meant the strengthening of FAO, resulting in the increased utilization of fatty acids. Limited studies have also shown that reduced plasma fatty acids were mainly caused by the FAO (39). Thus, our results from a population perspective validated the previous finding, which demonstrated FAO was a common disturbing metabolic pathway in mice exposed to benzene compared with controls (15, 40). In addition, FAO was found to sustain ATP levels to ensure cancer cell survival and proliferation, which was particularly relevant to the survival of leukemia cells (41, 42). In a case-control study on the association of plasma metabolites with AML carried out in a Chinese population, it was found that the precursors (oleic acid and palmitic acid) of the FAO pathway in plasma from AML were lower than controls, which supported our results in benzene-exposed workers (11). In addition, fatty acids are central to human health and disease because of their diverse biological roles (43). They have been reported to be dysregulated in many diseases such as type 2 diabetes (44, 45), Alzheimer’s disease (46), and AML (11). Because of their structural diversity and substantial taxonomic specificity, they have conferred biomarker property in organisms. To our knowledge, no absolute quantitative data are available regarding the exploration of the fatty acid levels in benzene-exposed workers and the association between fatty acids and benzene exposure. In this study, we highlighted that the levels of plasma fatty acids were significantly associated with benzene-induced hematotoxicity in benzene-exposed workers and mice. This finding reveals that the dysregulation of fatty acid metabolism participates in the occurrence and development of benzene-related blood diseases and that FAO was a major disturbing metabolic pathway.

The primary strength of this study was that we discovered 8 common fatty acids in benzene-exposed workers and mice that could be used as potential effector molecules associated with benzene-induced hematotoxicity. Extraordinary, absolute quantitative metabolomics was used to detect plasma metabolites, which will provide a direct reference range for plasma metabolite content in benzene-exposed workers. Secondly, our study also suggested that FAO might be the potential pathway for the early prediction of benzene-induced hematotoxicity. However, there were also some limitations in our study. Firstly, other possible covariates (such as personal habit, dietary, and income) might be missed, although the age, sex, BMI, and smoking and alcohol status had been considered. Secondly, our findings were observed in a cross-sectional design with a limited number of benzene-exposed workers and controls. Thus, the candidate metabolites in this study should be validated from an independent and larger sample. Finally, although an interesting dynamic change of fatty acids was verified in mice, further mechanistic investigations are needed in the future.

### Conclusions.

In summary, our results reveal that fatty acid metabolism was strongly reprogrammed after benzene exposure and FAO might be the major disturbing metabolic pathway in benzene-induced hematotoxicity. Early benzene exposure induced slight decreases in fatty acid levels, but with increasing benzene-induced hematotoxicity, a decompensated rise in fatty acids was found. These dynamic changes in metabolite levels might be the critical metabolic signatures of benzene-induced hematotoxicity. Eight identified plasma fatty acids (oleic acid, 10z-heptadecenoic acid, palmitoleic acid, DHA, myristoleic acid, dodecanoic acid, myristic acid, and linoleic acid) were significantly altered in both benzene-exposed workers and mice, and they mediated the association between benzene exposure and WBC reduction. This panel of differential fatty acids may be sensitive effector molecules that could contribute to early health screening for hematotoxicity in benzene-exposed populations.

## Methods

### Study subjects.

All subjects (Han Chinese, China, Asia) including benzene-exposed workers and healthy controls were recruited from the Research Center of Occupational Medicine, Peking University Third Hospital, Beijing, China, from December 2018 to February 2019. The benzene-exposed workers in this study were mainly engaged in painting work in an automobile repair shop, while the controls were office workers in the same enterprise. Subjects were excluded based on the following criteria: (a) subjects older than 50 years; (b) history of occupational exposure, such as carbon monoxide or formaldehyde; (c) history of smoking and alcohol consumption for more than 10 years; and (d) suffering from metabolic diseases, such as diabetes or gout. Finally, 162 subjects aged from 20–50 years, including 86 low-level benzene–exposed workers and 76 healthy controls, were enrolled.

### Sample collection.

Fasting venous blood (3 mL) were connected for routine blood and transaminase tests. Within 4 hours of blood sample collection, the samples were centrifuged at 1500*g* for 10 minutes at 4°C to separate plasma for metabolites measurement. Accordingly, middle segment urinary samples (10–15 mL) were obtained from subjects in the morning. Finally, 150 μL plasma and 2 mL urine were dispensed after centrifugation (4°C, 1500*g* for 10 minutes) and stored at –80°C until chemical analysis.

### Urinary benzenes metabolites detection.

Quantitative analysis of urinary benzene and xylenes metabolites was performed by an UPLC-MS/MS detectors. The sample preparation, derivatization protocols, and quality control were elaborated in Supplemental Methods. The parameters and methods of the instrument were set as shown in Supplemental Table 5. The raw data generated by UPLC-MS/MS were used for peak integration, correction, and quantitative analysis by QuanMET software (v1.0, Metabo-profile) for each metabolite. The urinary SPMA, tt-MA, 2-MHA, and 3-MHA levels were corrected by Cr, and the corresponding concentrations were presented in μg/g Cr.

### Routine blood detection.

The subjects were asked to maintain a normal diet and exercise within 3 days and fast overnight (>8 hours) before blood collection. Whole blood samples were analyzed for WBC, neutrophil, lymphocyte, platelet (PLT), HGB, and RBC by the Sysmex XS500i Automated Hematology Analyzer (Sysmex Corporation). ALT and aspartate transaminase (AST) in plasma were analyzed by the VITROS 5600 Integrated System (Johnson & Johnson Corporation).

### Plasma metabolomics analysis.

Plasma samples were used to assess individual metabolites, including amino acids, organic acids, amines, fatty acids, carbohydrates, and bile acids, with UPLC-MS/MS. The sample preparation and derivatization protocols were based on the method previously published, with minor modifications (17, 47, 48). The details of sample preparation and derivatization protocols were listed in Supplemental Methods. The instrumental parameters of the analysis are shown in Supplemental Table 6. A standard calibration solution with more than 300 standards at 7 different concentration levels was analyzed to construct the calibration curve. Peak annotation and quantitation were conducted by the TargetLynx application manager (Waters Corp.). Internal standards were added to the test samples to monitor analytical variations during the entire sample preparation and analysis process. The source and catalog number of all standards refer to the previous study (17).

### Animals and benzene treatment.

A total of 36 male C57BL/6J mice aged 4 weeks were purchased from Huafukang Bioscience Co. The benzene was purchased from Sigma-Aldrich (catalog 401765). By using the random number table, all mice were randomly allocated into 2 groups (control group, corn oil solvent, *n* = 18; 125 mg/kg × body weight benzene group, *n* = 18), and treated with 5 s.c. injections a week of corn oil solvent (COFCO Corporation, Q/JTYLY 0008S) or oil-benzene mixture for 15, 30, and 45 days. The body weight of the mice was obtained once a week during benzene exposure. The basis for the selection of exposure times and doses in animal models was described in the Supplemental Methods. Subsequently, the levels of WBC, RBC, HGB, and PLT were tested using a TEK-II mini automatic blood cell analyzer (Jiangxi Tekang Technology Ltd.). Furthermore, the detection of targeted metabolites was also achieved based on UPLC-MS/MS.

### Statistics.

The raw data files generated by UPLC-MS/MS were processed using the TMBQ software (v1.0, HMI, Shenzhen) to perform peak integration, calibration, and quantitation for each metabolite. To ensure data quality, manual examination and correction were performed. The Kolmogorov-Smirnov test was used to confirm the normal distribution of raw data. Mann-Whitney *U* test/Wilcoxon rank-sum test or Student’s *t* test (2-tailed) was performed to compare the differences in clinical indicators (routine blood and liver function parameters), plasma metabolites, and urinary benzene and xylenes metabolites between 2 groups in the cross-sectional study. Categorical variables (sex, smoking status, and alcohol consumption) were analyzed using the χ^2^ test. Data were presented using mean ± SD for normality data or median–interquartile range (M, IQR) for nonnormal data. Before the statistical analysis, significantly different plasma metabolomics and urinary benzene and xylenes metabolite data were log_10_ transformed to improve the normal distribution. The potential confounders of age, sex, BMI, smoking, and alcohol consumption were employed as covariates in the subsequent analyses. Partial correlation or Spearman analysis was used to investigate the correlations between either 2 variables. Linear regression models were used to test the associations among urinary benzene and xylenes metabolites, differential plasma metabolites, and blood cell counts. In this study, *P* < 0.05 (2-tailed) was considered the main threshold in all statistical tests for a statistically significant difference. The Benjamini-Hochberg (BH) method was performed to control the FDR, and *q* < 0.25 was used as a significant level for the multiple comparison correction (49). The bootstrap simple mediation model was used to further verify the intermediary role of differential plasma metabolites. To investigate the critical pathways involved in differential metabolites, we used the KEGG database and MSEA network for metabolite enrichment analysis and annotated key metabolic enzymes associated with differential metabolites. Statistical analyses were conducted using SPSS 20.0 and R (version 4.0.1).

### Study approval.

Human population studies and all animal experimental protocols were approved by the Ethical Committee of Capital Medical University (AEEI-2020-168). Written informed consent was also obtained from the subjects prior to the study.

## Author contributions

XG collected samples, analyzed data, and wrote the manuscript. LZ conducted animal experiments, analyzed data, and revised the manuscript. JW and WZ helped to collect samples and interpret the data. JR and YC collected samples. YZ collected samples, provided advice, and revised the manuscript. AG and YZ designed the study, interpreted the data, revised the manuscript, and supervised the research.

## Supplementary Material

Supplemental data

## Figures and Tables

**Table 1:**
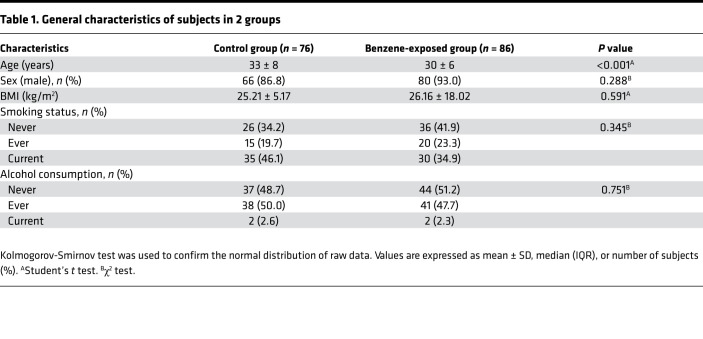
General characteristics of subjects in 2 groups

**Table 2:**
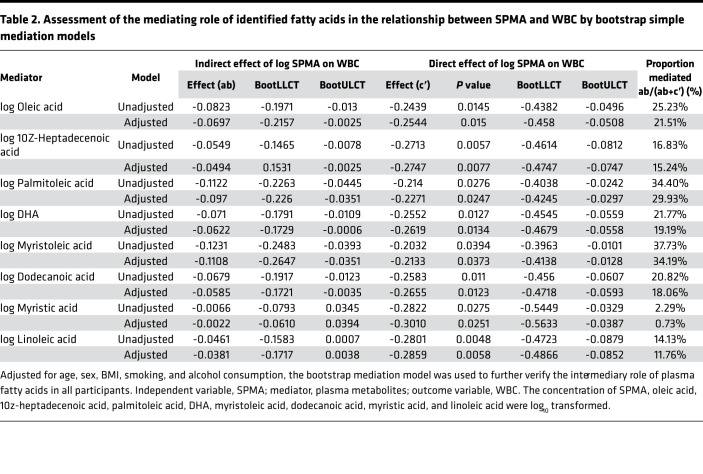
Assessment of the mediating role of identified fatty acids in the relationship between SPMA and WBC by bootstrap simple mediation models

## Version 1 01/25/2022

Electronic publication
