# Supplementary material for: Plasma metabolomics study reveals the critical metabolic signatures for benzene-induced hematotoxicity
Source: JCI Insight. 2022 Jan 25;7(2):e154999. doi: 10.1172/jci.insight.154999 (PMC8855792; doi:10.1172/jci.insight.154999)
Supplement: Supplemental data [file jciinsight-7-154999-s132.pdf]

Supplementary Materials

Plasma metabolomics study reveals the critical metabolic signatures for benzene-induced hematotoxicity

Xiaoli Guo,[1,2#] Lei Zhang,[1,2#] Jingyu Wang,[1,2] Wei Zhang,[1,2] Jing Ren,[1,2] Yujiao Chen,[1,2] Yanlin Zhang,[3*] and Ai Gao[1,2*]

[1.]Department of Occupational Health and Environmental Health, School of Public Health, Capital Medical University, Beijing 100069, P. R. China

[2.]Beijing Key Laboratory of Environmental Toxicology, Capital Medical University, Beijing 100069, P. R. China

[3.]Research Center of Occupational Medicine, Peking University Third Hospital, 49 North Garden Road, Haidian District, Beijing 100191, P. R. China

[#]Those authors contributed equally to this work.

Correspondence Authors: Prof. Ai Gao, Department of Occupational Health and Environmental Health, School of Public Health, Capital Medical University, Beijing 100069, P. R. China, Tel: 86-10-83911509 and Dr. Yanlin Zhang, Research Center of Occupational Medicine, Peking University Third Hospital, Beijing 100191, P.R. China.

Email: gaoai0980@163.com (A. Gao), zhangyanlin@bjmu.edu.cn (Y. Zhang).

Supplementary materials and methods

Chemicals and Reagents

All the 400 standards were obtained from Sigma-Aldrich (St. Louis, MO, USA), Steraloids Inc. (Newport, RI, USA) and TRC Chemicals (Toronto, ON, Canada). All the standards were accurately weighed and prepared in water, methanol, sodium hydroxide solution, or hydrochloric acid solution to obtain individual stock solution at a concentration of 5.0 mg/mL. Appropriate amount of each stock solution was mixed to create stock calibration solutions. Formic acid was of analytical grade and obtained from Sigma-Aldrich (St. Louis, MO, USA). Methanol (Optima LC-MS), acetonitrile (Optima LC-MS), and isopropanol (Optima LC-MS) were purchased from Thermo-Fisher Scientific (FairLawn, NJ, USA). Ultrapure water was produced by a Mill-Q Reference system equipped with a LC-MS Pak filter (Millipore, Billerica, MA, USA).

Urine benzene and xylenes metabolite detection

In this experiment, xevotq-s high performance liquid chromatography-tandem four-stage bar mass spectrometry (UPLC-MS/MS, Waters) was used to detect urine benzene and xylenes metabolites. Prior to analysis, take out urine samples and leave it on ice to thaw slowly. Then, the urine sample was centrifuged at 13,000 g at 10°C for 10 min. Subsequently, 80 μL standard and urine supernatant were taken, and 720 μL 10% formic acid solution was added to acidify (PH about 2). The solid-phase extraction column of Oasis MAX 96-well Plates was activated and balanced successively with 1mL methanol and 1ml deionized water, and then 800 μL acidified samples were added. Wash with 1 mL methanol solution containing 50 mM sodium acetate (5:95), elute the neutral substance with 1 mL methanol, and discard the eluent. 1ml methanol solution containing 2% formic acid was eluted for 2 times, combined with 2 times eluent and centrifuged for drying. The solution was

redisclosed with 160 μL 15 mM ammonium acetate solution and transferred to 350 μL 96-well plate. It was oscillated at 650 rpm for 10min at 10°C, then centrifuged at 4000 g for 10 min at 4°C, transferred 100 μL supernatant to another 96-well plate, and waited for detection. The parameters and methods of the instrument are set as shown in Table S5. There is reagent blank, system blank and system balance biological sample before and after sample analysis of each batch. The addition of these quality controls also monitors possible contamination and data quality during the analysis. In order to eliminate errors caused by the sequence of analysis process, samples to be tested were randomly measured according to group information. QC samples and blank samples are interspersed in the whole sample for testing. The raw data generated by UPLC-MS/MS will adopt QuanMET software (v1.0, Metabo-profile, Shanghai, China) perform peak integration, correction and quantitative analysis for each metabolite.

Plasma metabolomics analysis

Plasma sample used to assess individual metabolite including amino acids, organic acids, amines, fatty acids, carbohydrates, and bile acids which performed on an ultra-performance liquid chromatography coupled to tandem mass spectrometry (UPLC-MS/MS) system. Briefly, samples were thawed on ice-bath to diminish sample degradation. 25 μL of plasma was added to a 96-well plate and the plate was transferred to the Biomek 4000 workstation (Biomek 4000, USA). Ice cold methanol with internal standards was automatically added to each sample and vortexed for 5 min. Then the plate was centrifuged at 4000 g for 30 min (Allegra X-15R，USA). 30 μL of supernatant was transferred to a clean 96-well plate, and 20μL of freshly prepared derivative reagents was added to each well. The plate was sealed and carried out at 30°C for 60 min. After derivatization, 350 μL of ice-cold 50% methanol solution was added to dilute the sample and the plate was stored at -20°C

for 20 min and followed by 4000 g centrifugation at 4°C for 30 min. 135 μL of supernatant was transferred to a new 96-well plate with 15 μL internal standards in each well. Serial dilutions of derivatized stock standards were added to the left wells. Finally, the plate was sealed for UPLC-MS/MS analysis.

An ultra-performance liquid chromatography coupled to tandem mass spectrometry system (ACQUITY UPLC-Xevo TQ-S, USA) was used to measure the metabolites. The instrumental parameters of the analysis were set as shows in Table S5. A standard calibration solution with more than 300 standards at 7 different concentration levels were analyzed to construct the calibration curve. Peak annotation and quantitation were conducted by TargetLynx application manager (Waters Corp., Milford, MA, United States). Internal standards were added to the test samples in order to monitor analytical variations during the entire sample preparation and analysis processes.

Basis for selection of exposure time and dose in animal experiments

The hematopoietic system is a recognized target of benzene exposure. Long-term chronic benzene exposure could inhibit the renewal and differentiation of BM HSCs, ultimately resulting in altered hematology (1). To investigate the changes in metabolite profiles at different stages of benzene exposure-induced hematotoxicity, mice were treated with subcutaneous injections of 125 mg/kg of benzene for 15, 30 and 45 days. Respiratory inhalation and dermal contact are the two main pathways of benzene exposure in humans (2). Therefore, dynamic inhalation and subcutaneous injection are widely used in animal experiments. In the pre-experiments of our group, it was found that the ratio of mixed gas and pure gas in the dynamic inhalation device was unstable at low-level benzene, which might lead to the system error of benzene exposure concentration. In addition, real-time benzene exposure concentrations in the contaminated room were monitored by activated

carbon adsorption-CS2 desorption combined with gas chromatography, which is a complex and time-consuming procedure (http://www.nhc.gov.cn/wjw/pyl/201712/0c849c68aa9b48549056712aea6f97b9.shtml). Compared to dynamic inhalation, subcutaneous injection is convenient and allows the injection of accurate doses by calculation to mice. In addition, the choice of dose is critical to the success of a mouse model of benzene exposure-induced hematotoxicity. Previous literature on benzene exposure in mice indicated that subcutaneous administration of 150 mg/kg benzene for 30 days had been acknowledged by numerous researchers due to its stable and reproducible hematotoxicity (3, 4). Therefore, our current experimental dose was close to the previous studies, and a mouse model of benzene-induced hematotoxicity had been successfully constructed at this dose. In traditional toxicology, about 1/10 of the mouse life span (1-2 years) is considered a subchronic toxicity cycle. Therefore, the 30-day exposure period was widely used in in vivo studies in benzene exposure-induced hematotoxicity (4, 5). However, it is not clear whether there are dynamic changes in hematotoxicity and metabolic disorders due to benzene exposure during different exposure periods. Taken together, subcutaneous injections of 125 mg/kg benzene for 15, 30 and 45 days were selected in our subsequent animal experiments.

Table S1. Comparisons of urinary benzenes metabolites, whole blood cells, and liver function parameters between two groups

Effect indicators	Gender	Control group (n=76)			Benzene-exposed group (n=86)			p	q
		Mean ± SD	Median (IQR)	Min-Max	Mean ± SD	Median (IQR)	Min-Max		
Urinary benzene and xylene metabolites									
SPMA (μg/g Cr)	ALL	0.57 ± 0.92	0.12 (0.002-0.87)	0.001-5.22	0.97 ± 0.98	0.78 (0.14-1.45)	0.002-3.76	0.003[b]	0.012
	Male	0.58 ± 0.94	0.18 (0.001-0.76)	0.001-5.22	0.95 ± 0.93	0.82 (0.14-1.44)	0.002-3.76	0.005[b]	0.024
	Female	0.55 ±0.76	0.01 (0.002-1.45)	0.001-1.73	1.53 ± 1.46	1.13 (0.20-3.11)	0.09-3.69	0.126[b]	0.146
tt-MA (μg/g Cr)	ALL	290.59 ± 129.14	267.28 (218.51-334.07)	67.90-635.93	252.55 ± 153.95	249.86 (163.96-314.89)	58.53-1130.45	0.040[b]	0.085
	Male	267.84 ± 86.75	257.77 (217.07-321.82)	67.90-635.93	252.63 ± 156.30	246.04 (164.09-312.62)	58.53-1130.45	0.170[b]	0.170
	Female	459.94 ± 240.12	348.82 (334.97-525.00)	250.28-568.02	257.39 ± 122.92	262.45 (120.77-376.41)	116.19-362.33	0.059[b]	0.109
2-MHA (μg/g Cr)	ALL	229.11 ± 172.86	206.35 (98.93-334.65)	63.70-695.68	369.42 ± 789.24	178.91 (81.94-361.56)	40.45-1089.38	0.929[b]	0.413
	Male	233.70 ±170.49	221.32 (96.83-337.22)	63.70-628.64	382.23 ± 808.32	179.56 (85.26-378.10)	40.45-1089.38	0.855[b]	0.352
	Female	194.97 ± 197.06	119.91 (98.36-212.19)	85.23-695.67	215.10 ±241.220	138.54 (82.10-308.35)	42.70-178.91	0.926[b]	0.401
3-MHA (μg/g Cr)	ALL	475.80 ± 382.40	422.50 (155.20-757.45)	67.99-1377.10	577.29 ± 712.66	241.66 (135.42-855.74)	47.81-2048.83	0.804[b]	0.328
	Male	493.34 ± 398.28	425.58 (139.69-831.10)	67.99-1377.10	597.70 ± 726.06	276.36 (133.10-866.23)	47.81-2048.83	0.883[b]	0.364
	Female	345.19 ± 201.08	309.06 (159.33-497.13)	154.07-711.15	472.12 ± 711.34	206.93 (135.80-661.48)	143.49-241.66	0.480[b]	0.243
Blood parameters									
WBC ($*10^9$/L)	ALL	6.74 ± 1.54	6.58 (5.55-7.95)	4.57-12.21	6.10 ± 1.10	5.95 (5.36-6.97)	4.07-8.78	0.014[a]	0.049
	Male	6.76 ± 1.55	6.64 (5.58-7.97)	4.57-12.21	6.01 ± 1.01	5.91 (5.36-6.83)	4.07-8.17	0.005[a]	0.036
	Female	6.64 ± 1.53	6.27 (5.41-7.80)	4.74-9.40	6.55 ± 1.65	6.73 (4.80-7.97)	4.48-8.78	0.906[a]	0.388
NEUT ($*10^9$/L)	ALL	3.81 ± 1.21	3.54 (3.01-4.50)	2.02-8.51	3.76 ± 1.29	3.66 (2.84-4.35)	1.46-9.12	0.897[a]	0.376
	Male	3.80 ± 1.22	3.54 (3.01-4.50)	2.02-8.51	3.78 ± 1.31	3.66 (2.86-4.39)	1.46-9.12	0.948[a]	0.425
	Female	3.96 ± 1.17	3.73 (3.07-4.47)	2.70-6.42	3.49 ± 1.11	3.44 (2.59-4.47)	1.99-4.99	0.651[a]	0.267
LYMPH ($*10^9$/L)	ALL	2.31 ± 0.53	2.23 (1.86-2.74)	1.19-3.48	2.28 ± 0.56	2.18 (1.82-2.77)	1.31-3.83	0.641[a]	0.255
	Male	2.35 ± 0.53	2.43 (1.90-2.76)	1.19-3.47	2.29 ± 0.56	2.18 (1.86-2.78)	1.31-3.83	0.425[a]	0.219
	Female	2.03 ± 0.52	1.95 (1.73-2.15)	1.46-3.28	2.18 ± 0.68	2.10 (1.63-2.62)	1.45-3.33	0.724[a]	0.279

PLT (*10^9/L)	ALL	267.27 ± 56.69	265.00 (240.00-292.00)	155.00-461.00	250.29 ± 52.4	250 (217.25-278)	120.00-399.00	0.051[a]	0.097
	Male	267.21 ± 58.63	263.00 (238.50-292.50)	155.00-461.00	250.10 ± 52.61	251.50 (251.5-277.50)	120.00-399.00	0.084[a]	0.121
	Female	267.67 ± 42.32	42.32 (245.50-297.00)	178.00-325.00	252.83 ± 54.22	241.00 (205.50-303.25)	195.00-340.00	0.443[a]	0.231
HGB (g/L)	ALL	157.08 ± 13.10	159.00 (151.00-165.00)	123.00-188.00	158.70 ± 11.07	160.00 (153.00-165.00)	116.00-184.00	0.377[a]	0.206
	Male	160.03 ± 10.37	160.00 (154.00-166.50)	128.00-188.00	158.64 ± 10.22	160.00 (153.25-164.75)	116.00-178.00	0.751[a]	0.291
	Female	135.44 ± 10.66	133.00 (129.00-138.50)	123.00-160.00	159.50 ± 20.72	163.00 (163.00-178.75)	133.00-184.00	0.015[a]	0.061
RBC (*10^{12}/L)	ALL	5.16 ± 0.45	5.14 (4.95-5.40)	4.04-6.36	5.19 ± 0.37	5.21 (5.03-5.42)	4.03-5.90	0.300[a]	0.182
	Male	5.25 ± 0.38	5.19 (5.01-5.44)	4.31-6.36	5.20 ± 0.33	5.21 (5.03-5.41)	4.03-5.90	0.976[a]	0.437
	Female	4.51 ± 0.31	4.48 (4.29-4.78)	4.04-5.00	5.09 ± 0.67	5.20 (4.34-5.72)	4.20-5.86	0.099[a]	0.134
Liver function parameters									
AST (U/L)	ALL	22.87 ± 13.16	20.50 (17.00-26.00)	13.00-41.00	22.56 ± 9.78	21.00 (17.00-25.00)	13.00-44.00	0.778[a]	0.316
	Male	23.61 ± 13.81	21.00 (17.00-26.00)	13.00-41.00	22.73 ± 10.08	21.00 (17.00-25.00)	13.00-44.00	0.755[a]	0.303
	Female	17.33 ± 3.04	16.00 (14.50-20.50)	14.00-22.00	20.33 ± 3.56	20.50 (18.00-22.25)	15.00-26.00	0.154[a]	0.158
ALT (U/L)	ALL	29.85 ± 21.38	24.00 (15.00-37.00)	5.00-126.00	29.38 ± 23.13	23.50 (15.00-37.00)	7.00-170.00	0.837[a]	0.340
	Male	32.03 ± 21.66	27.00 (17.00-39.50)	8.00-126.00	39.50 ± 23.84	23.50 (15.00-37.00)	7.00-170.00	0.330[a]	0.194
	Female	13.89 ± 9.45	12.00 (8.00-15.50)	5.00-37.00	22.50 ± 7.58	22.50 (16.00-27.50)	13.00-35.00	0.034[a]	0.073

Normality distributions of all variables were checked by the Kolmogorov-Smirnov tests.

a: Student's t-test; b: Mann-Whitney U test.

$p<0.05$ indicates that the difference is statistically significant; q-value is adjusted p-value using FDR. Bold: q-value < 0.25.

Abbreviation: urinary benzene metabolite S-phenylmercapturic acid (SPMA) and trans,trans-muconic acid (tt-MA); xylene metabolite 2-methylhippuric acid (2-MHA) and 3-mercaptohexyl acid, 3-MHA; Routine blood , white blood cell (WBC), neutrophile granulocyte (NEUT), lymphocyte (LYMPH), blood platelet (PLT), hemoglobin (HGB), and red blood cell (RBC); Hepatic function index, aspartic transaminase (AST) and glutamic-pyruvic transaminase (ALT).

Table S2. Comparisons of 28 plasma differential metabolites levels between benzene-exposed workers and healthy controls (μmol/L)

Class	Metabolite	HMDB	Healthy control (n=76)		Benzene-exposed worker (n=86)		FC	p	q
			Median	IQR (P25-P75)	Median	IQR (P25-P75)			
SCFAs	Butyric acid	0000039	3.948	(2.969, 5.192)	4.710	(3.586, 6.026)	1.19	0.026	0.289
SCFAs	Isobutyric acid	0001873	4.530	(3.447, 6.862)	5.464	(4.191, 8.020)	1.21	0.043	0.328
Phenylpropanoic Acids	Phenyllactic acid	0000779	0.015	(0.008, 0.024)	0.019	(0.011, 0.030)	1.27	0.034	0.298
Peptides	Glycylproline	0000721	0.073	(0.070, 0.077)	0.076	(0.071, 0.079)	1.04	0.024	0.280
Organic Acids	Pyruvic acid	0000243	119.005	(90.081, 140.801)	93.989	(72.846, 122.038)	0.79	0.001	0.106
Organic Acids	cis-Aconitic acid	0000072	4.853	(4.260, 5.463)	5.276	(4.595, 5.962)	1.09	0.012	0.203
Organic Acids	Oxoglutaric acid	0000208	16.590	(11.099, 26.027)	13.196	(9.554, 19.838)	0.80	0.014	0.208
Fatty Acids	Oleic acid	0000207	1222.356	(983.106, 1506.540)	1076.129	(933.777, 1218.446)	0.88	0.001	0.106
Fatty Acids	10Z-Heptadecenoic acid	0060038	4.843	(3.603, 8.004)	3.900	(2.791, 5.423)	0.81	0.002	0.106
Fatty Acids	Palmitoleic acid	0003229	76.984	(49.210, 111.200)	54.566	(36.262, 80.827)	0.71	0.002	0.106
Fatty Acids	DHA	0002183	156.201	(116.529, 210.356)	130.661	(101.871, 158.341)	0.84	0.003	0.107
Fatty Acids	Myristoleic acid	0002000	0.227	(0.146, 0.390)	0.158	(0.090, 0.255)	0.70	0.003	0.107
Fatty Acids	Dodecanoic acid	0000638	0.754	(0.609, 1.045)	0.609	(0.504, 0.822)	0.81	0.004	0.107
Fatty Acids	Myristic acid	0000806	17.651	(13.217, 23.502)	15.048	(11.544, 18.239)	0.85	0.006	0.160
Fatty Acids	Linoleic acid	0000673	512.643	(445.400, 624.669)	462.359	(417.236, 534.033)	0.90	0.007	0.164
Fatty Acids	2-Butenoic acid	0010720	3.887	(3.019, 5.642)	4.629	(3.624, 6.226)	1.19	0.031	0.298
Fatty Acids	DPA	0006528	7.880	(6.099, 11.092)	7.063	(5.900, 8.570)	0.90	0.038	0.309
Fatty Acids	Eicosadienoic acid	0005060	185.059	(136.639, 236.277)	153.743	(124.776, 195.820)	0.83	0.044	0.328
Carboxylic acids	trans_Aconitic acid	0000958	6.665	(5.796, 7.675)	7.303	(6.442, 8.419)	1.10	0.014	0.208
Carbohydrates	Xylose	0000098	2.075	(1.726, 2.309)	1.829	(1.582, 2.155)	0.88	0.010	0.184
Carbohydrates	Threonic acid	0000943	1.121	(0.955, 1.321)	0.971	(0.756, 1.273)	0.90	0.034	0.298
Carbohydrates	Trehalose	0000975	18.177	(17.073, 19.514)	19.128	(17.294, 20.278)	1.04	0.037	0.309
Amino Acids	Homoserine	0000719	0.972	(0.838, 1.201)	1.126	(0.931, 1.298)	1.16	0.010	0.184

Amino Acids	alpha-Aminobutyric acid	0000452	0.669	(0.501, 0.889)	0.580	(0.432, 0.711)	0.87	0.017	0.229
Amino Acids	Asparagine	0000168	76.771	(70.801, 84.959)	80.472	(73.528, 88.920)	1.05	0.031	0.298
Amino Acids	Aminoadipic acid	0000510	1.9165	(1.032, 2.612)	1.338	(0.880, 1.980)	0.70	0.032	0.298
Amino Acids	Methionine	0000696	18.359	(15.633, 21.534)	19.764	(17.181, 23.078)	1.08	0.049	0.354
Alkylamines	Putrescine	0001414	0.051	(0.030, 0.073)	0.040	(0.017, 0.060)	0.78	0.023	0.280

Mann-Whitney-Wilcoxon (U-test) was performed to compare plasma differential metabolites levels between two group.

$p < 0.05$ indicates that the difference is statistically significant. q value is adjusted p-value using FDR. Bold: q value < 0.25.

Fold change (FC): the ratio of Exposeure/Control.

Table S3. Associations of urinary benzenes metabolites with whole blood cell and liver function indexes with adjusting age, smoking, drinking, and BMI in subjects

	Gender	log SPMA			log tt-MA			log 2-MHA			log 3-MHA		
		Adjusted β (95%CI)	p	q	Adjusted β (95%CI)	p	q	Adjusted β (95%CI)	p	q	Adjusted β (95%CI)	p	q
WBC	**Male**	**-0.33 (-0.52, -0.14)****	**0.001**	**0.008**	-0.61 (-1.44, 0.21)	0.143	0.229	-0.29 (-0.77, 0.2)	0.244	0.651	**-0.77 (-1.39, -0.15)***	**0.016**	0.128
	Female	-0.22 (-1.11, 0.66)	0.577	0.769	1.55 (-0.88, 3.98)	0.183	0.732	-0.38 (-2.39, 1.64)	0.681	1.362	0.99 (-1.72, 3.69)	0.43	1.048
NEUT	Male	-0.09 (-0.25, 0.08)	0.298	0.795	-0.1 (-0.83, 0.63)	0.787	0.787	0.03 (-0.41, 0.46)	0.899	0.899	-0.3 (-0.86, 0.25)	0.281	0.562
	Female	0.02 (-0.8, 0.83)	0.963	0.963	1.4 (-0.78, 3.59)	0.180	1.440	-0.4 (-2.21, 1.41)	0.629	2.516	0.59 (-1.89, 3.08)	0.603	2.423
LYMPH	Male	-0.005 (-0.09, 0.08)	0.904	1.033	-0.14 (-0.49, 0.21)	0.416	0.555	-0.15 (-0.36, 0.06)	0.167	1.336	**-0.3 (-0.57, -0.04)***	**0.025**	0.100
	Female	0.16 (-0.15, 0.46)	0.285	0.456	0.23 (-0.97, 1.43)	0.679	0.905	-0.08 (-0.99, 0.83)	0.848	1.131	0.16 (-1.1, 1.41)	0.278	0.516
PLT	Male	-2.5 (-10.68, 5.67)	0.546	0.728	-29.33 (-63.89, 5)	0.093	0.372	-11.32 (-32.74, 10.11)	0.298	0.477	-23.81 (-50.65, 3.02)	0.081	0.216
	Female	15.27 (-3.6, 34.14)	0.1	0.267	29.12 (-51.7, 109.93)	0.441	0.706	15.88 (-46.07, 77.82)	0.571	4.568	5.46 (-80.81, 91.73)	0.891	1.218
HGB	Male	-0.1 (-1.75, 1.56)	0.909	0.909	2.06 (-4.96, 9.08)	0.563	0.643	-0.48 (-4.81, 3.86)	0.828	0.946	-0.38 (-5.87, 5.11)	0.89	1.017
	Female	-0.53 (-11.11, 10.05)	0.912	1.042	21.8 (-14.73, 58.34)	0.213	0.568	6.3 (-23.29, 35.9)	0.645	1.720	25.28 (-11.7, 62.27)	0.159	1.793
RBC	Male	-0.03 (-0.08, 0.03)	0.291	1.164	0.22 (-0.01, 0.44)	0.064	0.512	0.08 (-0.07, 0.22)	0.315	0.420	0.09 (-0.09, 0.27)	0.326	0.522
	Female	0.01 (-0.27, 0.29)	0.268	0.536	0.38 (-0.64, 1.39)	0.429	0.858	-0.05 (-0.84, 0.74)	0.89	1.017	0.36 (-0.7, 1.41)	0.471	0.545
AST	Male	0.67 (-1.2, 2.53)	0.48	0.768	6.03 (-1.76, 13.81)	0.128	0.341	3.3 (-1.58, 8.18)	0.183	0.732	1.11 (-3.07, 5.29)	0.599	0.799
	**Female**	**1.64 (0.4, 2.87)***	**0.015**	0.120	-0.32 (-7.11, 6.48)	0.92	0.920	0.1 (-5.03, 5.24)	0.966	0.966	-1.7 (-8.64, 5.24)	0.597	0.116
ALT	Male	0.61 (-2.69, 3.91)	0.368	0.736	10.48 (-3.32, 24.29)	0.135	0.270	5.1 (-3.53, 13.74)	0.244	0.488	0.04 (-10.01, 10.09)	0.994	0.994
	**Female**	**3.95 (0.29, 7.62)***	**0.037**	0.148	1.71 (-16.45, 19.86)	0.838	0.958	-1.96 (-15.63, 11.71)	0.756	1.210	-1 (-19.84, 17.82)	0.907	0.179

Linear regression models of urinary benzenes metabolites with whole blood cell and liver function indexes after adjusting for age, BMI, smoking and alcohol consumption.

*p < 0.05; ** p < 0.01; q value is adjusted p-value using FDR. Bold: q value < 0.25.

Table S4. Correlation analysis of differential metabolites with hematological parameters and liver function parameters in all participants

	WBC	NEUT	LYMPH	PLT	HGB	RBC	AST	ALT
Oleic acid	0.32**	0.18*	-0.02	0.17*	0.25**	0.23**	0.07	0.16*
10Z-Heptadecenoic acid	0.32**	0.17*	0.03	0.12	0.17*	0.12	0.08	0.19*
Palmitoleic acid	0.35**	0.18*	0.03	0.17*	0.20*	0.13	-0.06	0.08
DHA	0.262**	0.11	0.00	0.04	0.24**	0.21**	0.01	0.11
Myristoleic acid	0.32**	0.12	0.06	0.14	0.11	0.05	-0.08	0.03
Dodecanoic acid	0.25**	0.08	0.00	0.04	0.04	-0.07	-0.13	-0.06
Myristic acid	0.36**	0.18*	0.09	0.06	0.20*	0.13	0.06	0.17*
Linoleic acid	0.24**	0.19*	-0.06	0.23**	0.22**	0.22**	0.07	0.17*

** Significant correlation at the 0.01 level (two-tailed).

* Significant correlation at the 0.05 level (two-tailed).

Table S5. UPLC-TQMS instrument parameters settings (Urine benzene and xylenes metabolite detection)

UPLC	
Column	ACQUITY UPLC UPLC@ HSS T3 1.8μm 2.1 mm × 100 mm
Column Temp. (°C)	40
Sample Manager Temp. (°C)	10
Mobile Phases	A=water+15 mM Ammonium acetate; B=acetonitrile
Gradient Conditions	0-2 min (3-5% B), 2-3 min (5-10% B), 3-5 min (10-30% B), 5-6.5 min (30-40% B),6.5-7 min (40-15% B), 7-7.5 min (15-10% B), 7.5-8 min (10-3% B), 7-7.5 min (15-10% B)
Flow Rate (mL/min)	0.3
Injection Vol. (μl)	5
Flow Rate (mL/min)	0.3
Injection Vol. (μl)	5
MASS SPECTROMETER	
Capillary (kv)	3.5 (ESI-)
Source Temp (°C)	150
Desolvation Temp (°C)	550
Desolvation Gas Flow (L/Hr)	1000

Table S6. UPLC-TQMS instrument parameters settings (Plasma metabolomics analysis)

UPLC	
Column	ACQUITY UPLC BEH C18 1.7 μM VanGuard pre-column (2.1×5 mm) and ACQUITY UPLC BEH C18 1.7 μM analytical column (2.1 × 100 mm)
Column Temp. (°C)	40
Sample Manager Temp. (°C)	10
Mobile Phases	A=water with 0.1% formic acid; and B=acetonitrile/IPA (90:10)
Gradient Conditions	0-1 min (5% B), 1-12 min (5-80% B), 12-15 min (80-95% B), 15-16 min (95-100%B), 16-18 min (100%B), 18-18.1 min (100-5% B), 18.1-20 min (5% B)
Flow Rate (mL/min)	0.4
Injection Vol. (μl)	5
MASS SPECTROMETER	
Capillary (kv)	1.5 (ESI+), 2.0 (ESI-)
Source Temp (°C)	150
Desolvation Temp (°C)	550
Desolvation Gas Flow (L/Hr)	1000

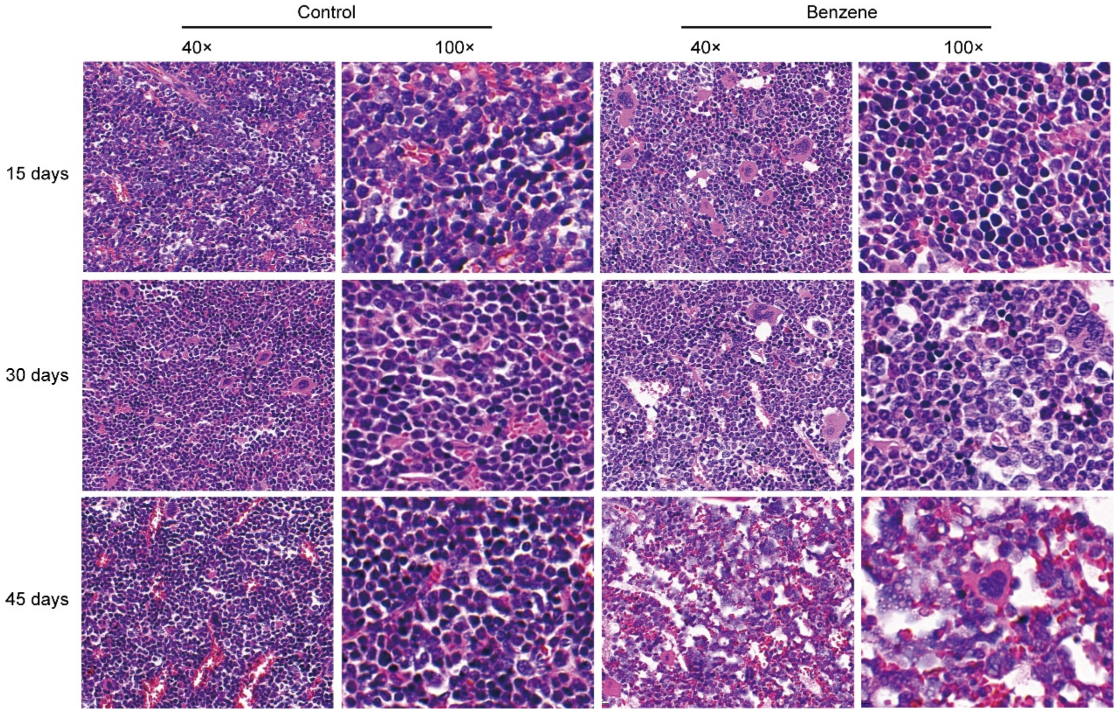

Figure S1. Representative images of H&E staining of femur tissues in mice exposed to benzene for 15, 30 and 45 days (Magnification of 40× and 100×).

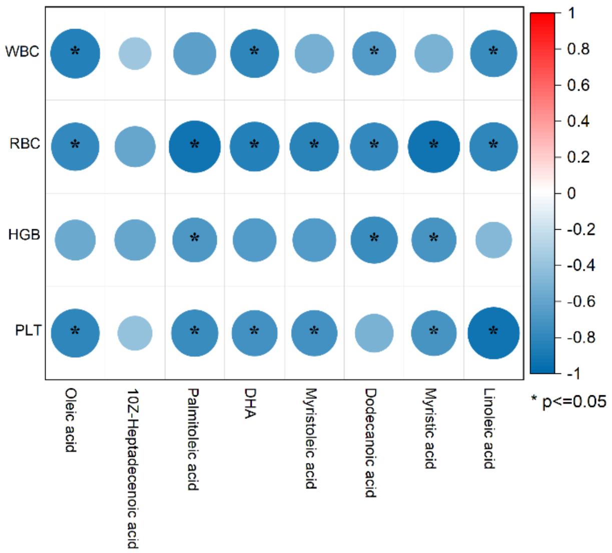

Figure S2. Correlation of blood parameters (WBC, RBC, HGB. PLT) with differential fatty acids (Oleic acid, 10Z_Heptadecenoic acid, Palmitoleic acid, DHA, Myristoleic acid, Dodecanoic acid, Myristic acid, Linoleic acid) in mice after 45 days of benzene exposure. * p < 0.05.

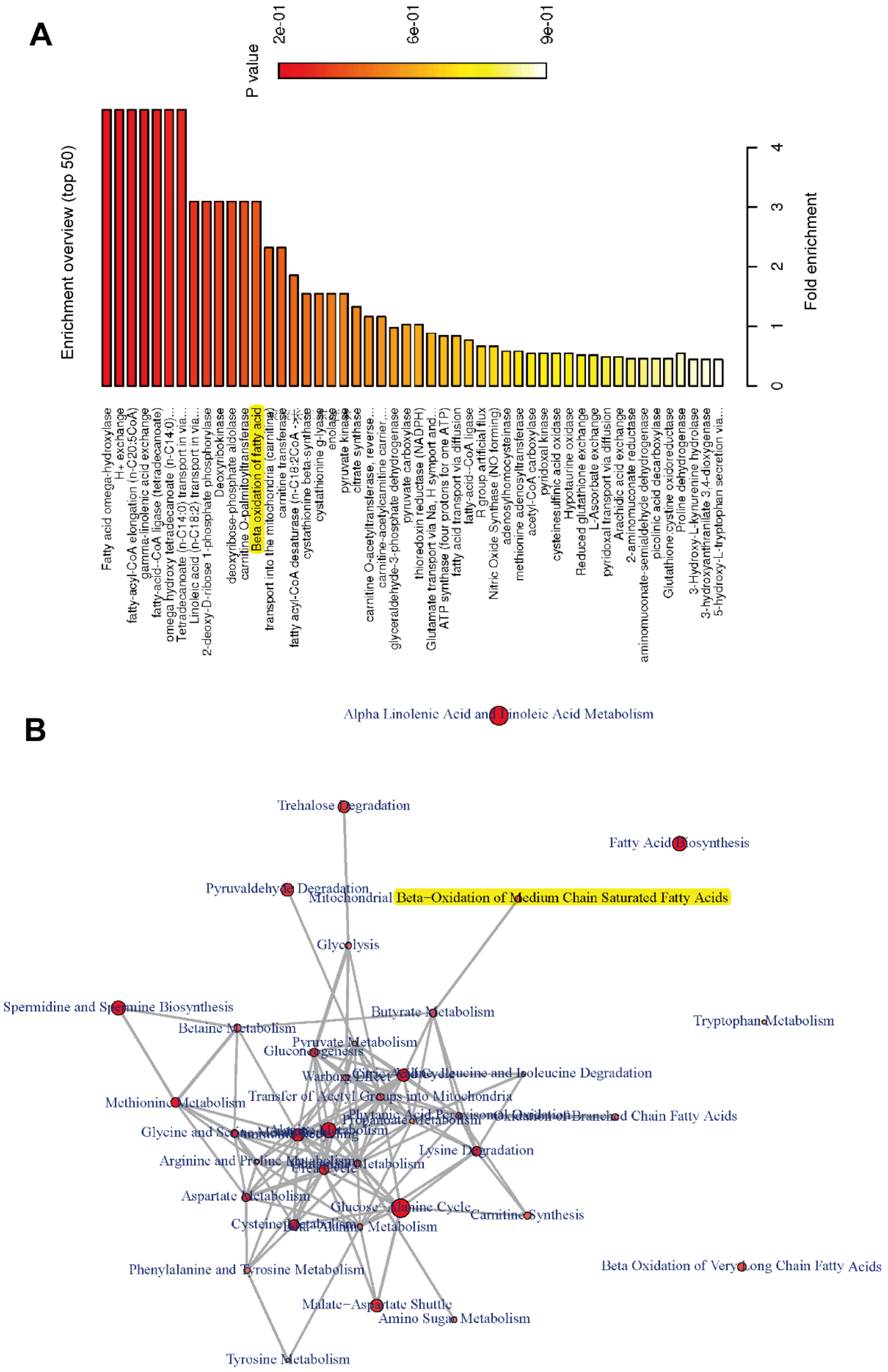

Figure S3. Metabolite enrichment analysis. (A) Enrichment barplot based on KEGG database. (B) MSEA network.

Reference

1. Lamm SH, and Grunwald HW. Benzene exposure and hematotoxicity. *Science.* 2006;312(5776):998–9.
2. Weisel CP. Benzene exposure: an overview of monitoring methods and their findings. *Chem Biol Interact.* 2010;184(1-2):58–66.
3. Sun R, Xu K, Ji S, Pu Y, Man Z, Ji J, et al. Benzene exposure induces gut microbiota dysbiosis and metabolic disorder in mice. *Sci Total Environ.* 2020;705:135879.
4. Sun R, Xu K, Ji S, Pu Y, Yu L, Yin L, et al. Toxicity in hematopoietic stem cells from bone marrow and peripheral blood in mice after benzene exposure: Single-cell transcriptome sequencing analysis. *Ecotoxicol Environ Saf.* 2021;207:111490.
5. Liang B, Chen Y, Yuan W, Qin F, Zhang Q, Deng N, et al. Down-regulation of miRNA-451a and miRNA-486-5p involved in benzene-induced inhibition on erythroid cell differentiation in vitro and in vivo. *Arch Toxicol.* 2018;92(1):259–72.
